# Photophysical Properties of Donor-Acceptor Stenhouse Adducts and Their Inclusion Complexes with Cyclodextrins and Cucurbit[7]uril

**DOI:** 10.3390/molecules25214928

**Published:** 2020-10-24

**Authors:** Liam Payne, Jason D. Josephson, R. Scott Murphy, Brian D. Wagner

**Affiliations:** 1 Department of Chemistry, University of Prince Edward Island, Charlottetown, PE C1A 4P3, Canada; lpayne@upei.ca; 2 Department of Chemistry and Biochemistry, University of Regina, Regina, SK S4S 0A2, Canada; jjose031@uottawa.ca (J.D.J.); scott.murphy.uregina@gmail.com (R.S.M.)

**Keywords:** DASAs, photoswitches, supramolecular chemistry, fluorescence, cyclodextrins, cucurbiturils, host-guest inclusion, fluorescence enhancement

## Abstract

Donor-acceptor Stenhouse adducts (DASAs) are a novel class of solvatochromic photoswitches with increasing importance in photochemistry. Known for their reversibility between open triene and closed cyclized states, these push-pull molecules are applicable in a suite of light-controlled applications. Recent works have sought to understand the DASA photoswitching mechanism and reactive state, as DASAs are vulnerable to irreversible “dark switching” in polar protic solvents. Despite the utility of fluorescence spectroscopy for providing information regarding the electronic structure of organic compounds and gaining mechanistic insight, there have been few studies of DASA fluorescence. Herein, we characterize various photophysical properties of two common DASAs based on Meldrum’s acid and dimethylbarbituric acid by fluorescence spectroscopy. This approach is applied in tandem with complexation by cyclodextrins and cucurbiturils to reveal the zwitterionic charge separation of these photoswitches in aqueous solution and the protective nature of supramolecular complexation against degradative dark switching. DASA-M, for example, was found to form a weak host-guest inclusion complex with (2-hydroxypropyl)-γ-cyclodextrin, with a binding constant K = 60 M^−1^, but a very strong inclusion complex with cucurbit[7]uril, with K = 27,000 M^−1^. This complexation within the host cavity was found to increase the half-life of both DASAs in aqueous solution, indicating the significant and potentially useful stabilization of these DASAs by host encapsulation.

## 1. Introduction

The term “photoswitch” refers to a broad palette of compounds capable of undergoing some reversible change in structure, conformation, charge, etc. in response to the absorption of light. This reversibility allows for the application of photoswitches as binary state switches, analogous to turning a light switch on or off [1]. Electronic excitation can induce bond rotation and cleavage, among other processes [2]. The ability to convert molecules between forms using light presents a wide range of applications ranging from photocontrol of enzyme activity [3], to tissue-specific drug release in vivo [4], to super-resolution fluorescence microscopy techniques [5,6]. Common examples of such photoswitchable molecules include azobenzenes [7], spiropyrans [8], hemithioindigos [9], and cyanines [10].

Donor-acceptor Stenhouse adducts (DASAs) are molecular photoswitches that can be unidirectionally controlled using visible light. This class of push-pull olefins consists of a triene skeleton with opposing electron-donating and electron-withdrawing groups (EDG and EWG, respectively) [1]. Generally, the EDG is limited to a secondary amine or some *N*-alkyl aniline derivative, whereas the EWG is largely customizable and can be selected to tune the absorption profile of these intensely colored compounds [11]. The legacy of DASAs spans 150 years, beginning with the discovery of Stenhouse salts in 1870 [12]. Recent renewal of interest in DASA compounds was sparked by a 2014 paper published by Read de Alaniz and co-workers [11]. Previous contributions from Honda [13], D’Arcy [14] and Šafár [15], and subsequent contributions from Read de Alaniz [16] and Feringa [17] have firmly established these molecules as a novel class of visible light-activated photoswitches that are synthetically accessible in high purity and yield.

**Figure 1:**
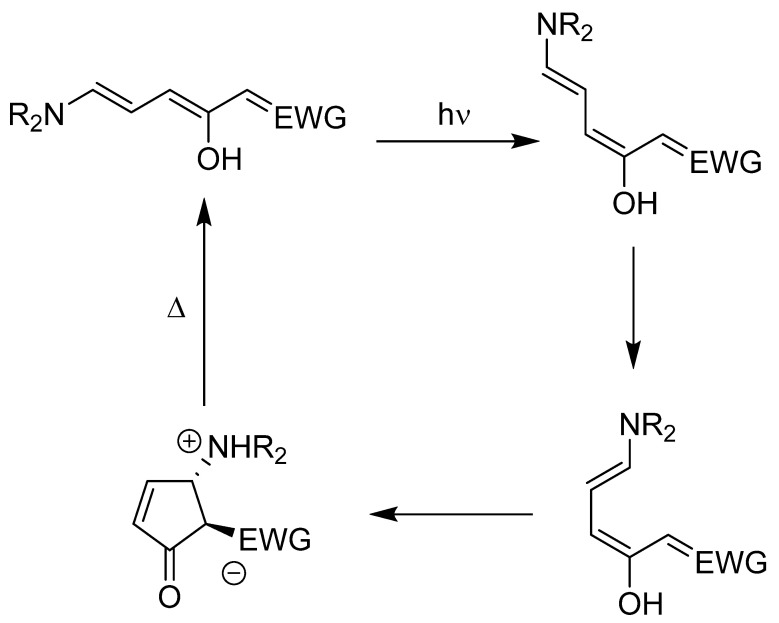
Photoswitching mechanism of donor-acceptor Stenhouse adducts (DASAs). Electronic excitation of the linearized triene via absorption of a photon promotes a series of rotations, culminating in an electrocyclization. The cyclopentenone form thermally reverts to the triene form.

The delocalization of the electron cloud across the extended conjugation of DASAs produces a prominent π→π* absorption band in the visible light range, giving intense color to these solids [17,18]. Additionally, delocalization reduces the double bond character linking conjugated carbon atoms, decreasing the energy barrier for rotation and thus bestowing photoswitchability [19]. The DASA photoswitching mechanism is relatively complex and has been mostly elucidated through a combination of experimental and computational studies [20,21,22,23,24,25]: following electronic excitation with visible light, a series of rotations followed by a conrotatory 4π-electrocyclization converts the linear triene form into a closed cyclopentenone form (Figure 1). Essentially, the colored “on” state switches to a colorless “off” state. Interestingly, reversion to the linearized triene from the cyclopentenone photoisomer occurs only thermally and cannot be photoinduced.

**Figure 2:**
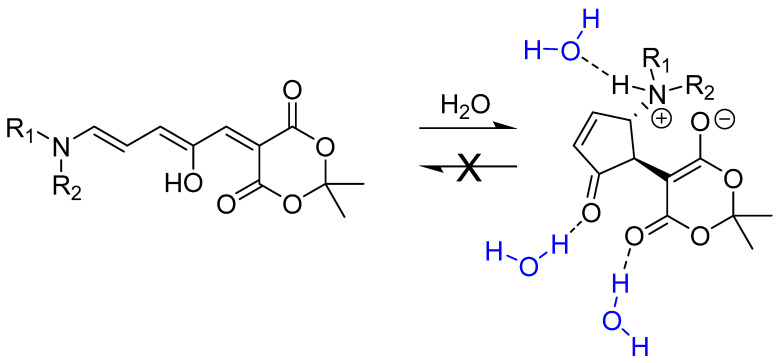
Water-mediated “dark switching” of a DASA, as proposed by Wang et al. [31]. Favorable hydrogen bonding with the zwitterionic cyclopentenone form leads to irreversible stabilization.

By blending their unique photochemistry and synthetic modularity, DASAs can be customized to fit a breadth of applications in both non-living and biological systems. Published applications include selective release of drug cargo from DASA-based micelles [26], submicron photolithography [27], and sensors for amines [28], metal cations [29,30], and changes in pH [30]. Intriguingly, Helmy et al. [11] found that polar protic solvents cause irreversible cyclization of DASAs to a zwitterion of the cyclopentenone form, even in the absence of light. This deleterious process is problematic for applications of DASAs in aqueous media, as the photoswitch becomes permanently locked in the colorless form. Recently, Wang et al. [31] have demonstrated that hydrogen bonding between solvent molecules and the carbonyl and ammonium moieties of the cyclopentenone form leads to DASA “dark switching” (Figure 2).

Unfortunately, few approaches have been found to successfully mitigate irreversible dark switching in polar protic solvents. Encapsulation of a DASA by a macrocycle to form a supramolecular inclusion complex in aqueous solution could potentially minimize dark switching, as (i) inclusion provides protection of the guest from interaction with water solvent molecules (all solvent molecules are typically ejected from the cavity upon guest inclusion), including hydrogen bonding with the DASA [32], and (ii) guests in inclusion complexes generally have lowered rotational freedom [33], physically preventing the steps required for the DASA to cyclize. This technique was used in the first reported stabilization of a DASA in its linear form in water by Saha et al. [34], wherein a Pd(II)-based molecular vessel was employed.

The use of non-toxic, commercially available host macrocycles such as cyclodextrins (CDs) [35] and cucurbiturils (CBs) [36] offers a more accessible approach to aqueous stabilization of DASAs. As these hosts are known to bind polymethine cyanine dyes with similar structures to DASAs [37,38,39], they were selected as candidates for this investigation. To the best of our knowledge, no prior works have investigated the propensity of CDs and CBs to bind DASAs and reduce dark switching. Supramolecular encapsulation of a guest molecule causes a local change in polarity, restricts molecular motion, and often results from favorable intermolecular interactions, all of which can significantly influence the energy and efficiency of relaxation by fluorescence emission [33,40,41,42]. Accordingly, fluorescence spectroscopy is effective for quantifying the strength of supramolecular associations in terms of the equilibrium binding constant, determined by way of fluorescence titration [40]. The highly conjugated nature of DASAs makes them excellent candidates for study using fluorescence spectroscopy; yet, publications reporting on the fluorescence of this photoswitch are sparse and limited to organic and mixed aqueous solvents [30,43]. Therefore, the use of fluorescence to investigate complexation of DASAs by CDs and CBs presents an opportunity to characterize their photophysical properties and probe their electronic structure.

The aims of this study are fourfold: (i) use UV-visible and fluorescence emission spectroscopy to characterize two DASAs in terms of fluorescence quantum yield and polarity sensitivity; (ii) to determine the binding constants for inclusion of these DASAs into cyclodextrin and cucurbituril host molecules; (iii) to assess the utility of this supramolecular complexation for preventing or slowing the dark switching of these photoswitches; (iv) combine results from spectroscopic and supramolecular studies to gain insight into the electronic structure of DASAs in polar protic media. It is paramount that a process for alleviating the cyclization of DASAs in water be developed in order for these photoswitches to have useful applications in biological settings. This project thus harmonizes the convenient and robust self-assembly of supramolecular chemistry with the high sensitivity of fluorescence spectroscopy in the investigation of donor–acceptor Stenhouse adducts.

## 2. Results and Discussion

### 2.1. Characterizing DASAs as Fluorophores

**Figure 3:**
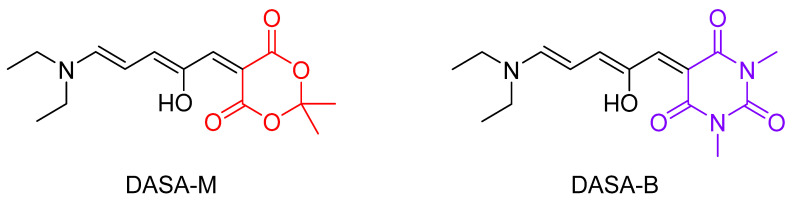
Chemical structure of photoswitches DASA-M and DASA-B.

Photoswitches DASA-M and DASA-B (Figure 3) were prepared according to procedures developed by Helmy et al. [11,16] (see Supplementary Materials). These two specific DASAs were selected as they are well-reported in the literature and their susceptibility to undergo dark switching in polar protic solvents has been studied [31]. DASAs with electron-withdrawing groups of different potencies were purposely selected such that observed differences in photophysical properties might be used to rationalize models for their electronic structure [18].

**Figure 4:**
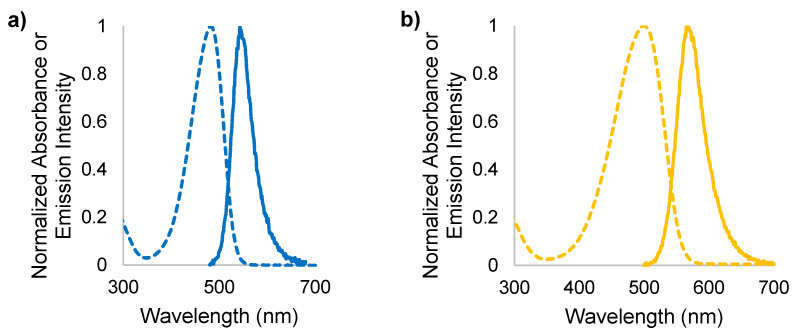

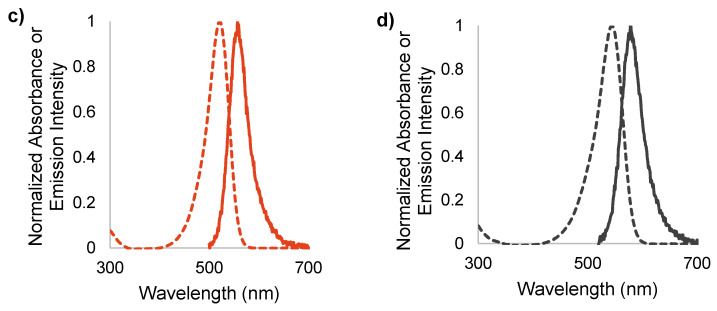
Absorption (- - -) and fluorescence emission (—) profiles of (**a**) DASA-M in water; (**b**) DASA-B in water; (**c**) DASA-M in EtOH; (**d**) DASA-B in EtOH.

The absorption and emission profiles of both DASAs were measured in nanopure water and ethanol. As lustrous solids, these photoswitches give intensely colored solutions with extraordinarily large molar extinction coefficients. Their absorption spectra are significantly red-shifted (i.e., >60 nm) in EtOH relative to those in water (Figure 4). This is unsurprising, as DASAs are known to display negative solvatochromism, meaning that bathochromic shifting occurs in decreasingly polar solvent systems. This phenomenon is the result of the excited singlet state having a smaller dipole moment than the ground state of DASAs [44], which means that the excited state is less destabilized in a lower polarity environment than the ground state. Since the ground state energy is increased more than the excited state, the energy gap between them is decreased, and the result is bathochromic (i.e., red) shifting of λ_A,max_ with decreasing solvent polarity (as clearly seen in Table 1).

Despite their strong absorption of visible light, both DASA-M and DASA-B exhibit weak fluorescence, with quantum yields (Φ_F_) of (2.5 ± 0.1) × 10^−4^ and (12 ± 2) × 10^−4^ in water, respectively (Table 2). Values of Φ_F_ for both photoswitches were determined using fluorescein in 0.1 M NaOH as a standard [45]. As radiative relaxation is in direct competition with photoswitching via rotational relaxation, it is not surprising that these molecules do not fluoresce strongly. The short length of the conjugated π system is another limiting factor [46]. However, DASA-B has a significantly higher fluorescence quantum yield than does DASA-M; this will be addressed below. In addition to the quantum yield, the polarity sensitivity factor (PSF) was measured for each DASA. The PSF was developed in our UPEI group [42] as a method to quantify the sensitivity of the emission intensity of a fluorophore to local polarity. The PSF is determined as the ratio of the integrated fluorescence emission spectrum in EtOH to that in water, corrected for absorbance (*cf.* Equation (3) in the Experimental section). PSF values are directly related to the ratio of the fluorescence quantum yield of the fluorophore in EtOH to that in water.

These results shown in Table 1 and Table 2 are interesting. Based on the difference in magnitude of the Stokes shifts in water and EtOH (Table 1), the relative energies of the electronic states involved in absorption and fluorescence are clearly sensitive to environmental polarity. The Stokes shift for both DASAs is significantly larger in water than in ethanol, a result of a much larger red-shifting of the absorption spectrum as compared with the fluorescence spectrum. This is consistent with the ground state being more polar than the excited state (resulting in the negative solvatochromism as discussed above), and hence more sensitive to medium polarity. The fluorescence quantum yield of the DASAs in water is seen to depend on the EWG, with the value for DASA-B being nearly 5 times larger than that for DASA-M. In addition, the degree of sensitivity of the DASA emission to solvent polarity is also dependent on the substitution, with the PSF values of DASA-M and DASA-B determined to be 1.37 ± 0.26 and 1.01 ± 0.13, respectively (Table 2). Furthermore, this means that despite the red-shift in the fluorescence maximum in ethanol vs. water, DASA-M is actually more fluorescent in ethanol. In the case of DASA-B, the fluorescence efficiency is roughly the same in the two solvents. Given the significant red-shifting of the emission in EtOH relative to water (i.e., a decreased excited state-ground state energy gap), the rate of internal conversion (i.e., nonradiative decay competing with fluorescence) will be larger in ethanol and hence the fluorescence quantum yield should decrease, according to the energy gap law [40]. Further, this assumes that the rate of photoswitching is similar in both solvents. Nonetheless, the PSF value of 1.37 in the case of DASA-M indicates that fluorescence is in fact more efficient in EtOH for this DASA. This observed increase in DASA-M fluorescence efficiency as polarity is decreased, and the higher fluorescence quantum yield of DASA-B relative to DASA-M will be addressed in the discussion below, in terms of the delocalization of their electronic structures.

**Figure 5:**
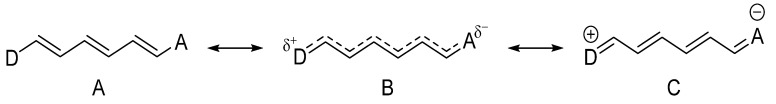
Possible electron distributions of DASAs. As the strength of the donor and acceptor groups increases, polarization increases, shifting from **A** (neutral triene form) to **B** (fully delocalized form) to **C** (zwitterionic triene form). Adapted from Refs. [47,49].

A rationalization of the interesting DASA photophysics described in the previous paragraph may be attributed to their push-pull nature, as explained by Bublitz et al. [47], who depict a simple conjugated chain with electron-donating and -accepting termini. In Figure 5, structures **A** and **C** are interchangeable via resonance, whereas **B** is a completely delocalized intermediate between these two contributors. If the donor and acceptor are weak, the DASA will take on an electron distribution most like **A**. Systems with increasing donor and acceptor strength polarize the π cloud, giving **B** or even **C** with potent push–pull groups. As **C** exhibits the maximum possible charge separation, the excited electronic state is virtually guaranteed to have decreased polarity, resulting in negative solvatochromism [47]. In polar solvents, structure **C** is stabilized, but the contribution from **B** is increased as the solvent becomes less polar (i.e., EtOH). Because this intermediate form has complete delocalization of electron density, excitation yields minimal changes in bond order and gives only weak vibronic interactions within the excited singlet manifold in accordance with the Franck–Condon Principle. As a result, absorption and emission bands become narrowed and their intensities increase in nonpolar environments, as has been shown previously for similar dipolar polyene compounds [48,49]. This increase in intensity due to increased Franck-Condon factors in less polar solvent (i.e., EtOH) clearly contributes towards the increased emission intensity of DASA-M in ethanol compared to water (PSF = 1.37), as structure **B** in Figure 5 would be most prevalent, and these solvent effects well explain the observed PSF larger than 1 for this DASA. Additionally, as the polar nature of DASAs decreases upon electronic excitation, the contributions of solute-solvent interactions to the stabilization of this state are minimized, giving undramatic solvatofluorochromism relative to the negative solvatochromism of these compounds [47,48,49].

This explanation is completely congruent with the observed photophysics of both DASA-M and DASA-B and suggests that these photoswitches take on a highly dipolar (i.e., charge-separated) form in polar solvents. The greater quantum yield of DASA-B in highly polar aqueous solution can also be reasoned based on this model, as a result of substituent effects. Since dimethylbarbituric acid is a strong electron-withdrawing group, this photoswitch may resemble **C** even more so than DASA-M. If this is the case, the bond orders of the conjugated chain are near true double bonds, meaning the barrier for rotation is relatively high. This hinders the ability of DASA-B to undergo photoswitching, increasing the relative efficiency of radiative relaxation. This is an important result, as DASAs are seldom depicted as their polarized resonance contributor **C**. The polarization of the conjugated system within DASAs imparts similar characteristics to previously reported polymethine dyes [49,50], meaning that they may share functionalities and applications.

### 2.2. Comparative Binding by Cyclodextrins and Cucurbiturils

As discussed, the use of commercially available, non-toxic host macrocycles is imperative to establishing supramolecular chemistry as a way to reduce DASA dark switching in aqueous applications. (2-hydroxypropyl)-γ-cyclodextrin (HP-γ-CD) was chosen as the model cyclodextrin host, as it is more water soluble than native CDs and caused the greatest fluorescence enhancement of all hosts in the hydroxypropylated series (Figure S3). Likewise, cucurbit[7]uril (CB[7]) was selected as the model cucurbituril host, as it is relatively soluble in water in comparison to other sizes of CBs [51], and has a compatible cavity size for these DASA guests.

As both DASA-M and DASA-B are polarized in the ground state, their emission is expected to be affected by the reduced polarity of the host cavity. Additionally, the hindered switching from the triene to the cyclized form due to limited rotational freedom when encapsulated is anticipated to increase the relative efficiency of fluorescence. A fluorescence titration measures how the emission of a fluorophore is enhanced or suppressed (F/F_0_) as a function of the concentration of a macrocyclic host ([H]). This allows for the equilibrium binding constant K to be determined, quantifying the formation of a host–guest inclusion complex. Assuming a 1:1 association between HP-γ-CD or CB[7] and the DASAs, a fluorescence titration should produce a curve given by Equation (1) [40,52]. The linearity of a double reciprocal plot of 1/(F/F_0_ − 1) vs. 1/[H] is commonly used to verify the validity of this assumption [40].

(1) $$\frac{F}{F_{0}}=\left(\frac{F_{\infty }}{F_{0}}-1\right)\frac{K\left[H\right]}{K\left[H\right]+1}+1$$

**Figure 6:**
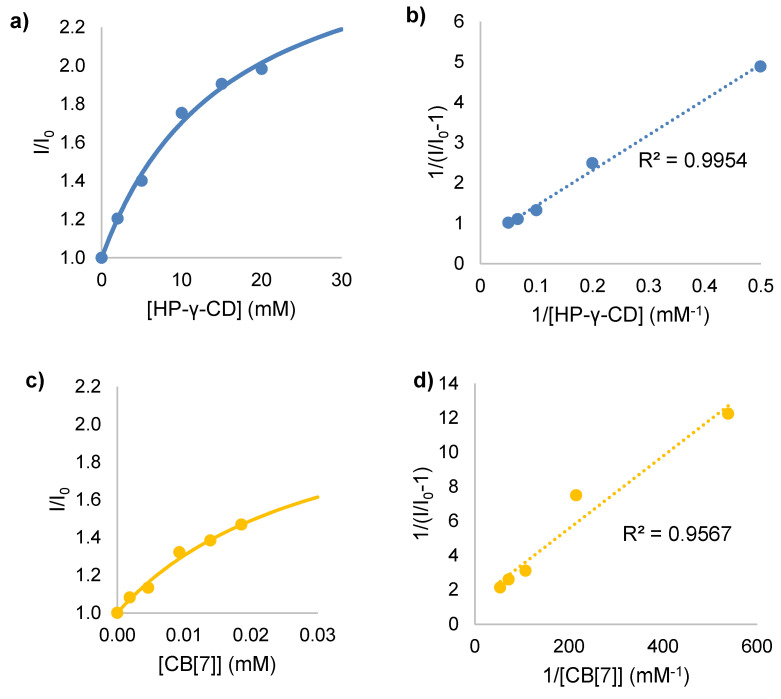
Fluorescence titration plots for DASA-M inclusion complexation: (**a**,**b**) HP-γ-CD titration curve and double reciprocal plot; (**c**,**d**) CB[7] titration curve and double reciprocal plot.

Multiple fluorescence titration experiments were conducted for DASA-M and DASA-B with both HP-γ-CD and CB[7]. Representative trials for DASA-M, including fitted titration curves and double-reciprocal plots, are shown in Figure 6. It should be noted that all absorption and fluorescence spectra were red-shifted with increased molar extinction coefficients in the presence of either macrocycle. This observation is consistent with the reverse solvatochromism of the DASAs, as the interior cavity of CDs and CBs are less polar than water. As prolonged exposure to a light source quickens the switching of DASAs to the colorless form, only narrow spectra were measured, and changes in intensity were used for calculations, as I/I_0_ ≈ F/F_0_. Emission intensities were recorded at 550 and 570 nm for DASA-M and DASA-B, respectively. The resultant titration curves and double reciprocal plots confirmed the formation of 1:1 host-guest inclusion complexes between HP-γ-CD and CB[7] and DASA-M and DASA-B.

Non-linear least squares fitting was performed on all titration data using an in-house program, allowing for binding constants to be determined (Table 3). For complexation of DASA-M and DASA-B by HP-γ-CD, K = 60 ± 3 M^−1^ and 39 ± 14 M^−1^, respectively. The magnitudes of these binding constants are not overly large, reflecting only a modest affinity of the HP-γ-CD cavity for binding the DASA guests. Maximum I/I_0_ values observed for DASA-M and DASA-B approached 2. Hydrogen bonding is a driving factor for the assembly of supramolecular complexes involving cyclodextrins; however, there are few opportunities for this interaction with the triene form of DASAs. Thus, inclusion is likely driven mainly by hydrophobic effects. The presence of added methyl groups in the structure of DASA-B increases steric bulk and worsens the potential for hydrogen bonding, possibly accounting for the slightly lower binding constant.

Analogous analyses were performed to extract binding constants for inclusion of each DASA within CB[7]. Interestingly, both values increased by nearly three orders of magnitude, with K = (2.7 ± 0.4) × 10^4^ M^−1^ and (8.9 ± 4.5) × 10^4^ M^−1^ for DASA-M and DASA-B, respectively. Maximum I/I_0_ values were approximately 1.6 and 1.9 for DASA-M and DASA-B, respectively. Clearly, different factors mediate inclusion within cucurbiturils and cyclodextrins. Ion-dipole interactions with the portals of negative charge fostered by CBs enables these hosts to stabilize positively charged guests [53,54]. Typically, this is observed for the conjugate acids of amines; however, strong binding of quaternary ammonium compounds and iminium ions has also been reported [55]. Here, the high affinity between DASA-M and DASA-B and the cavity of CB[7] suggests that both DASAs are in a highly charge separated state (structure **C** in Figure 5) in water, creating an iminium moiety that is drawn to the negative carbonyl groups of CB[7]. It is likely that the DASA guest is completely included within the CB[7] cavity, with the cationic end interacting the carbonyl oxygens of the CB[7] portal, and the rest of the guest residing within the central cavity, given such large binding constants. Furthermore, given that the value of K for DASA-B is nearly 3.3× larger than that of DASA-M, it is likely that this photoswitch exhibits stronger iminium cation character due to the increased electron-accepting ability of its dimethylbarbituric acid moiety. This is in agreement with the higher fluorescence quantum yield observed for DASA-B compared to DASA-M, which was also explained in the previous section as arising from the increased contribution of structure **C** in Figure 5 in the case of the former. It should be noted, however, that the coefficient of variation associated with this value is 51%, meaning this result has large uncertainty. Fluorescence titrations are well-suited for determining binding constants up to 10^4^ M^−1^, but are known to struggle with precision above this magnitude; this is mainly due to the error involved in using minimal masses of host molecules in combination with the exceptional sensitivity of fluorescence spectroscopy. To circumvent this issue, Liu et al. [56] have developed a “competition” method by which the subject of the study competes with a known reference to bind with CBs, allowing accurate and precise measurements of binding constants in excess of 10^12^ M^−1^. This approach should be considered for future studies of DASA-CB inclusion complexes. However, even with these relatively qualitative values of the binding constants for CB[7], the incredibly large values, together with the quantum yields and PSF results, provide strong evidence that DASAs exhibit significant charge separation in polar solvents like water.

An important finding of these experiments is that the magnitude of fluorescence enhancement (i.e., I/I_0_) was greater than the PSF of either DASA when encapsulated by HP-γ-CD or CB[7]. This indicates that the observed enhancement of fluorescence cannot be solely attributed to the lowered polarity of the CD cavity. From the perspective of the quantum yield, there is less competition between fluorescence and photoswitching or dark switching when DASAs are bound by a macrocycle, suggesting that the restricted motion within these architectures limits conversion from the DASA triene to its colorless “off” state. Thus, HP-γ-CD and CB[7] must offer a degree of protection from dark switching by physically blocking interactions between the DASAs and the surrounding water molecules and preventing intramolecular rotation.

### 2.3. Dark Switching within Supramolecular Complexes

Both HP-γ-CD and CB[7] demonstrate good abilities to encapsulate DASA-M and DASA-B in aqueous solution. It is therefore essential to evaluate the effect of such complexation on the rate of dark switching in aqueous solution. This was easily assessed by periodically measuring the absorbance of DASA solutions in the presence and absence of each macrocycle and analyzing the data based on first-order decay kinetics.

**Figure 7:**
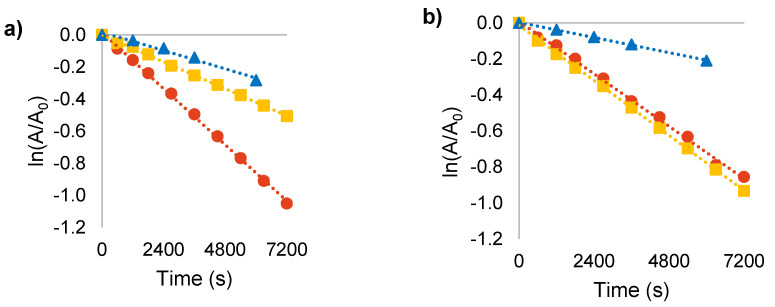
Dark switching with no host (●), 20 mM HP-γ-CD (■), or 2 mM CB[7] (▲): (**a**) semi-log plot of DASA-M dark switching kinetics; (**b**) semi-log plot of DASA-B dark switching kinetics.

The semi-log decay profiles (Figure 7a,b) demonstrate that dark switching of both DASA-M and DASA-B in aqueous solution is significantly reduced upon formation of supramolecular host-guest complexes, as the slopes directly correspond to the first-order switching rate constants. Interestingly, both HP-γ-CD and CB[7] are capable of sustaining the triene form of DASA-M in water, whereas DASA-B is only stabilized by CB[7]. This is in agreement with the lower binding constant between HP-γ-CD and DASA-B; however, some stabilization was expected. The increased bulk of the dimethylbarbituric acid moiety relative to Meldrum’s acid may result in different orientations of DASA-M and DASA-B residing within the cavity of HP-γ-CD, causing disparity in the degree to which interactions with water molecules are blocked upon encapsulation.

Remarkably, CB[7] was found to significantly extend the longevity of DASA-M and DASA-B in water, increasing their half-lives by a factor of 3.1 and 3.5, respectively (Table 4). Considering the modest improvement offered by HP-γ-CD, these results confirm that the driving forces for inclusion of DASAs into CDs and CBs are vastly different and support the notion that DASAs take on a dipolar electronic distribution in water. Notably, the dark switching of DASA-B is slower than DASA-M when both are complexed by CB[7]. As the electron-withdrawing group of DASA-B is stronger than that of DASA-M, the former will take on a stronger cationic nature, and thus DASA-B is bound more strongly by the CB[7] as evidenced by the larger binding constant. Thus, the prevalence of an iminium moiety lends to the favorable encapsulation of DASAs by CB[7], whereas the ability to form hydrogen bonds is the mediating factor for inclusion within HP-γ-CD.

These results are extremely promising, as they showcase the ability of supramolecular architectures to provide defense against DASA dark switching in water. While both cyclodextrins and cucurbiturils are able to bind these photoswitches and slow their conversion to the colorless state, it is evident that the exceptional ability of cation-binding CB[7] macrocycles to encapsulate DASAs is indispensable to the infinite stabilization of these molecules in polar protic media.

## 3. Materials and Methods

### 3.1. Chemicals and Instrumentation

For synthesis of DASA-M, 2,2-dimethyl-1,3-dioxane-4,6-dione, 2-furaldehyde, and diethylamine were purchased from Sigma-Aldrich Canada (Oakville, ON, Canada). For preparation of DASA-B, 1,3-dimethylpyrimidine was purchased from Sigma-Aldrich, and 2-furaldehyde and diethylamine were purchased from Alfa-Aesar (Haverhill, MA, USA). Additionally, tetrahydrofuran and dichloromethane were purchased from Fisher (Waltham, MA), hexanes and methanol were purchased from Sigma-Aldrich Canada, and absolute ethanol was purchased from Commercial Alcohols. For supramolecular studies, (2-hydroxypropyl)-γ-cyclodextrin and cucurbit[7]uril hydrate were purchased from Sigma-Aldrich. Reagents were used as received without further purification.

Water was collected from a Thermo Scientific Barnstead Easypure II water purification system. In-house deionized water was also used. The concentrations of the DASA stock solutions were in the range of 2 to 10 µM.

UV-visible measurements were performed on a Varian Cary 50 Bio UV-Visible Spectrophotometer and fluorescence spectroscopy experiments were conducted using a HORIBA PTI (Photon Technology International, Birmingham, NJ, USA) QuantaMaster400 Fluorimeter. NMR spectra were measured on a Bruker Avance Spectrometer (Billerica, MA, USA) (300 MHz) or a Varian Mercury plus spectrometer (Palo Alto, CA, USA) (300 MHz) in CDCl_3_ and processed using the TopSpin 4.7 software package.

### 3.2. Spectroscopic Measurements

All samples prepared for analysis by UV-visible absorption and/or fluorescence emission spectroscopies were prepared in 1 cm × 1 cm quartz cuvettes sealed with Teflon stoppers or rubber septa, as required. For absorption studies, all measurements were made at ambient room temperature and at a 1 cm path length. For fluorescence studies, all measurements were made at 25 °C. All spectra were recorded with band passes set to 1 nm, but emission slits were varied to give band passes between 1 and 2 nm depending on the specific experiment.

### 3.3. Quantum Yield

The value of Φ_F_ is easily calculated for a fluorescent candidate by comparing its spectroscopic properties with a known reference standard excitable at the same wavelength [41]. Prior to spectroscopic measurements, samples were purged with argon gas for roughly 5 min; however, DASAs showed no propensity for quenching of emission by O_2_ gas and Φ_F_ values were unchanged when samples were not purged. Excitation wavelength, bandpass, and scanning range were kept identical for the sample, reference standard, and solvent blanks. Integrated areas (F) were calculated for all emission spectra and solvent blanks were subtracted from their respective sample spectra. Φ_F_ values were measured in at least triplicate for both DASAs:

(2) $$\phi_{F}=\phi_{F,R}\left(\frac{F}{F_{R}}\right)\left(\frac{A_{R}}{A}\right){\left(\frac{n}{n_{R}}\right)}^{2}$$

### 3.4. Polarity Sensitivity Factor (PSF)

Solutions of DASAs were prepared in both nanopure water and absolute ethanol and each sample was purged with argon gas for approximately 5 min to prevent quenching by oxygen. Ideally, solutions were made such that the absorbance at the excitation wavelength was kept within the range of 0.2–0.4; however, lower absorbance values were used when necessary to prevent inner filter effects in the red-shifted ethanol solutions. UV-visible absorption and fluorescence emission spectra were measured for both the DASA solutions and their respective solvent blanks. For emission measurements, the excitation wavelength, bandpass, and scanning range were kept constant for both samples. Integrated areas were calculated for all emission spectra and solvent blanks were subtracted from their respective sample spectra. The PSF was measured in at least triplicate for both DASAs [42]:

(3) $$\mathrm{PSF}=\frac{F_{\mathrm{EtOH}}}{F_{H_{2}O}}\times \frac{A_{H_{2}O}}{A_{\mathrm{EtOH}}}$$

### 3.5. Fluorescence Titrations and Binding Constants

As DASAs undergo rapid switching to a colorless form in polar protic solvents, it was imperative that all measurements be performed in minimal time. A three-step “fast dilution” method was developed: (i) an aliquot of an aqueous DASA stock solution was used to dissolve a certain mass of the host molecule, giving a stock containing the DASA and a known concentration of host; (ii) various host concentrations were quickly achieved by mixing precise volumes of the host-absent and host-containing DASA stocks; and (iii) the fluorescence emission of each sample was measured over a 20 nm range centered about λ_F,max_. Solvent blanks were also measured for each trial and corresponding absorptions, intensities, and integrated areas were subtracted from the related sample measurements.

Based on the measured emission intensities at the selected wavelength, plots of I/I_0_ (measured at λ_F,max_) as a function of host concentration were made. This fluorescence titration data was fit using an in-house nonlinear least squares fitting program to extract the binding constant K using Equation (1), assuming a 1:1 host-guest ratio. Double reciprocal plots were also constructed to verify the 1:1 stoichiometry.

## 4. Conclusions

The fascinating photochemistry of photoswitches makes them suitable for a wide range of applications, and donor-acceptor Stenhouse adducts are no exception. This work successfully lays a foundation for using fluorescence spectroscopy in the study of these interesting molecules. Markedly, the measured photophysical parameters of DASAs (i.e., negative solvatochromism, Stokes shift in water and EtOH, quantum yield, and polarity sensitivity factor) were used to justify a model for the electronic distribution of these push-pull olefins and relate this to their ability to switch between triene and cyclopentenone forms. The interesting photophysical behavior of these two DASAs was explained based on solvent and substituent effects on this electron distribution model. This is highly important for DASA research, as the conceptualization of their structure as belonging on a spectrum of delocalization is key to application-tailored design. As discussed, this also highlights commonalities between DASAs and other well-studied polymethine dyes (e.g., cyanines).

Furthermore, this investigation has demonstrated that complexation of DASAs within cyclodextrins and cucurbiturils, namely HP-γ-CD and CB[7], can significantly lengthen their half-lives in aqueous solution by physically protecting them from interactions with solvent molecules and reducing rotational freedom. As CDs and CBs are highly biocompatible, they are superior alternatives to metal-based architectures. Moreover, the ability of the selected host macrocycles to enhance DASA fluorescence upon binding has shed light on the interesting electronic structure of these species and corroborates the model predicted based on their photophysical properties. Chiefly, the spectral red-shifting and extraordinarily favorable binding of DASA-M and DASA-B to CB[7] compellingly indicates that these photoswitches exhibit a highly dipolar, zwitterionic structure with significant charge separation in polar protic media.

As a relatively novel class of photoswitches with unique unidirectional photocontrol, DASAs represent an exciting and ever-growing field of research. With the deepened understanding of their photochemistry, electronic structure, and suitability for host inclusion acquired from this project, future studies should aim to elucidate more clearly the effect of environmental polarity on DASA polarization and how supramolecular encapsulation could be tailored to immortalize these compounds in aqueous solution.

## Figures and Tables

**Table 1:** Stokes shift parameters of DASA-M and DASA-B in water and EtOH.

	DASA-M		DASA-B	
Parameter	Water	Ethanol	Water	Ethanol
λ_A,max_ (nm)	482	521	500	544
λ_F,max_ (nm)	543	557	567	578
Stokes Shift (nm)	61	36	67	34
Stokes Shift (cm^−1^)	2331	1241	2344	1081

**Table 2:** Red shifts, quantum yield and polarity sensitivity of DASA-M and DASA-B.

Photophysical Property	DASA-M	DASA-B
Absorption spectrum red shift * (nm)	39	44
Absorption spectrum red shift * (cm^−1^)	1553	1618
Emission spectrum red shift * (nm)	14	11
Emission spectrum red shift * (cm^−1^)	463	335
Quantum yield in water (Φ_F_)	2.5 × 10^−4^	12 × 10^−4^
Polarity sensitivity factor (PSF)	1.37	1.01

* Absolute value of difference between peak maxima in water and in ethanol.

**Table 3:** Equilibrium binding constants for the host-guest complexation of DASA-M and DASA-B by HP-γ-CD and CB[7] hosts.

	K (M^−1^)	
Host	DASA-M	DASA-B
HP-γ-CD	60	39
CB[7]	27000	89000

**Table 4:** First-order rate constants for dark-switching and related half-lives in free solution and in the presence of hosts.

	DASA-M		DASA-B	
Condition	k (10^−5^ s^−1^)	τ_½_ (Min.)	k (10^−5^ s^−1^)	τ_½_ (Min.)
no host	14.6 ± 0.2	79 ± 1	12.1 ± 0.5	95 ± 2
20 mM HP-γ-CD	7.0 ± 0.1	165 ± 2	14.5 ± 2.5	81 ± 14
2 mM CB[7]	4.8 ± 0.4	242 ± 19	3.5 ± 0.1	330 ± 7

## Supplementary Materials

The following are available online. DASA compounds synthesis and characterization; NMR of DASA-M: Figures S1 and S2; fluorescence enhancement of DASA emission spectra by CDs and CB[7]: Figures S3 and S4; photographs of DASA solvatochromism in absence (Figure S5) and presence (Figure S6) of host. Reference [57] are cited in the supplementary materials.
