# Supplementary material for: Photophysical Properties of Donor-Acceptor Stenhouse Adducts and Their Inclusion Complexes with Cyclodextrins and Cucurbit[7]uril
Source: Molecules. 2020 Oct 24;25(21):4928. doi: 10.3390/molecules25214928 (PMC7662831; doi:10.3390/molecules25214928)
Supplement: Supplementary file 1 [file molecules-25-04928-s001.pdf]

molecules

Supplementary Material

Photophysical Properties of Donor-Acceptor Stenhouse Adducts and Their Inclusion Complexes with Cyclodextrins and Cucurbit[7]uril

Liam Payne [1], Jason D. Josephson [2], R. Scott Murphy [2] and Brian D. Wagner [1,*]

[1] Department of Chemistry, University of Prince Edward Island, Charlottetown, PE C1A 4P3, Canada; lpayne@upei.ca
[2] Department of Chemistry and Biochemistry, University of Regina, Regina, SK S4S 0A2, Canada; jjose031@uottawa.ca (J.D.J.); scott.murphy@uregina.ca (R.S.M.)
* Correspondence: bwagner@upei.ca; Tel.: 1-902-628-4351

Contents

1. Synthetic Methods S2

2. DASA Characterization S4

3. Fluorescence Enhancement from CDs and CB[7] S6

4. Solvent- and Host-Based Negative Solvatochromism S7

References S8

1. Synthetic Methods

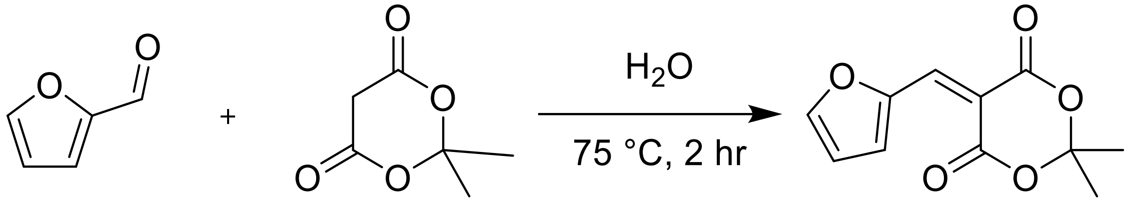

5-(Furan-2-ylmethylene)-2,2-dimethyl-1,3-dioxane-4,6-dione:

2-Furaldehyde (3.3 mmol) was pipetted into 10 mL H_2O with stirring. Once mixed, 2,2-dimethyl-1,3-dioxane-4,6-dione (3.3 mmol) was added. The yellow mixture was heated to 75 °C for 2 hours while stirring gently. The faint yellow mixture transitioned to an opaque green-brown suspension. After cooling to room temperature, the brown precipitate was collected via vacuum filtration through a Whatman #1 filter paper. The product was washed with 50 mL H_2O to remove soluble impurities. This gave 2.3 mmol (70%) of 5-(furan-2-ylmethylene)-2,2-dimethyl-1,3-dioxane-4,6-dione as a yellow-brown solid. NMR spectra match previously reported data [1].

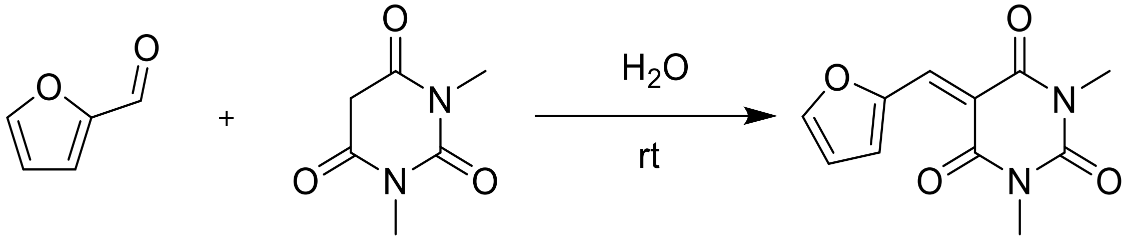

5-(Furan-2-ylmethylene)-1,3-dimethylpyrimidine-2,4,6(1*H*,3*H*,5*H*)-trione:

1,3-Dimethylpyrimidine-2,4,6(1*H*,3*H*,5*H*)-trione (10 mmol) was added to 40 mL water and stirred. To this suspension was added 2-furaldehyde (10 mmol), and stirring was continued overnight at room temperature, forming a yellow precipitate. Vacuum filtration was used to collect the precipitated solid, which was then washed twice with 30 mL cold H_2O. The precipitate was dissolved in 75 mL dichloromethane and washed with 30 mL saturated aqueous $NaHSO_3$, 30 mL H_2O, 30 mL saturated aqueous $NaHCO_3$, and 30 mL brine. The organic phase was dried over $MgSO_4$, then gravity filtered. The solvent was stripped by rotary evaporation to give 8.6 mmol (86%) of 5-(furan-2-ylmethylene)-1,3-dimethylpyrimidine-2,4,6(1*H*,3*H*,5*H*)-trione. NMR spectra match previously reported data [2].

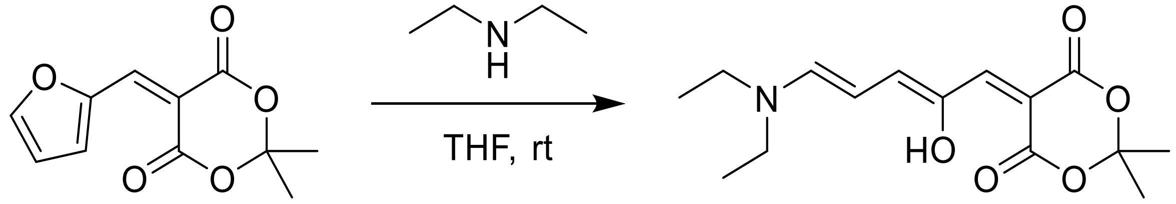

5-((2*Z*,4*E*)-5-(Diethylamino)-2-hydroxypenta-2,4-dien-1-ylidene)-2,2-dimethyl-1,3-dioxane-4,6-dione (DASA-M):

5-(Furan-2-ylmethylene)-2,2-dimethyl-1,3-dioxane-4,6-dione (1.8 mmol) was added to 4.5 mL tetrahydrofuran and stirred. Diethylamine (1.8 mmol) was added dropwise, instantly giving an opaque magenta mixture. The mixture was stirred at room temperature for 10 minutes, then cooled in an ice bath for 20 minutes, causing a magenta precipitate to appear. The 5-((2*Z*,4*E*)-5-(Diethylamino)-2-hydroxypenta-2,4-dien-1-ylidene)-2,2-dimethyl-1,3-dioxane-4,6-dione was collected via vacuum filtration through a Whatman #1 filter paper and the retentate was washed with

60 mL ice-cold diethyl ether. This gave 0.83 mmol (46%) DASA-M. NMR spectra match previously reported data [3].

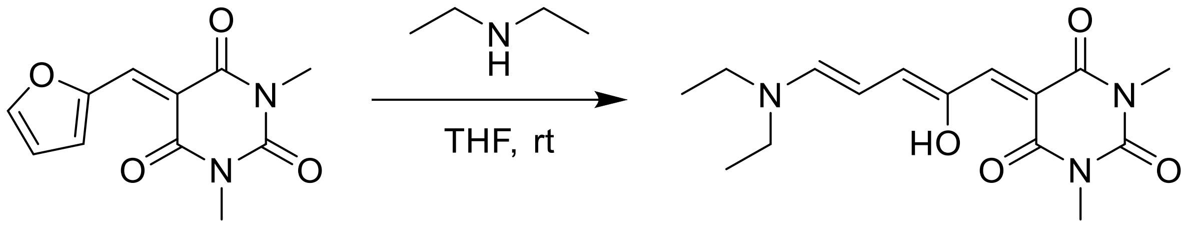

5-((2*Z*,4*E*)-5-(Diethylamino)-2-hydroxypenta-2,4-dien-1-ylidene)-1,3-dimethyl-pyrimidine-2,4,6(1*H*,3*H*,5*H*)-trione (DASA-B):

5-(Furan-2-ylmethylene)-1,3-dimethylpyrimidine-2,4,6(1*H*,3*H*,5*H*)-trione (1.0 mmol) was added to 10 mL tetrahydrofuran and stirred. Diethylamine (1.0 mmol) was added to the suspension, and the solution rapidly became pink, then dark purple. The mixture was stirred at room temperature for 30 minutes. Cold *n*-hexane was added to the reaction mixture to precipitate 5-((2*Z*,4*E*)-5-(diethylamino)-2-hydroxypenta-2,4-dien-1-ylidene)-1,3-dimethyl-pyrimidine-2,4,6(1*H*,3*H*,5*H*)-trione, which was then obtained as dark purple crystals by vacuum filtration. This gave 0.67 mmol (67%) DASA-B. NMR spectra match previously reported data [4].

2. DASA Characterization

DASA-M

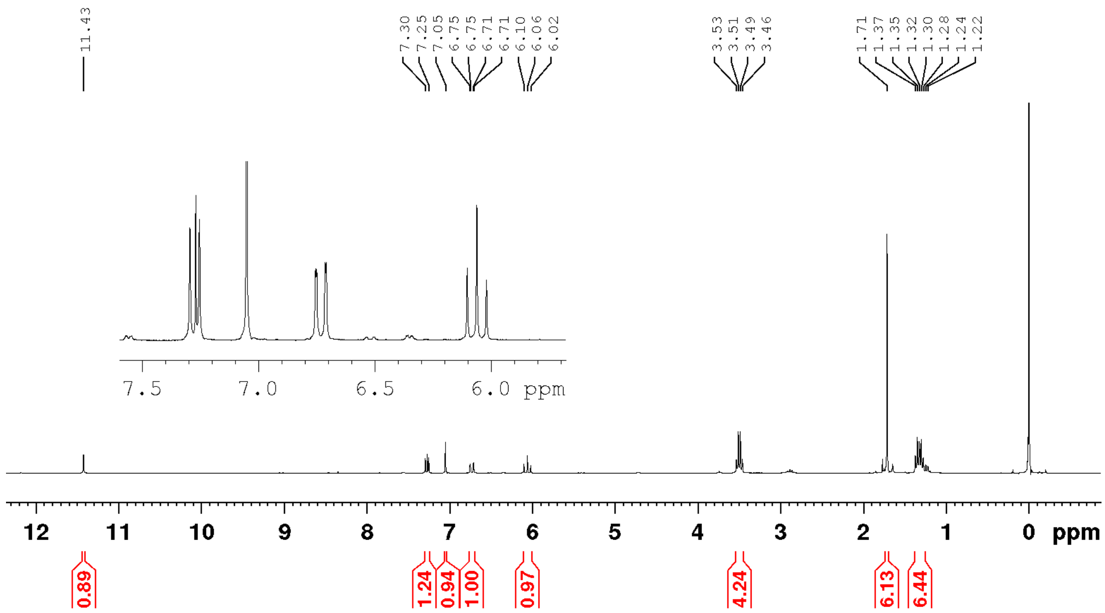

Figure S1. ^{1}H NMR spectrum of DASA-M.

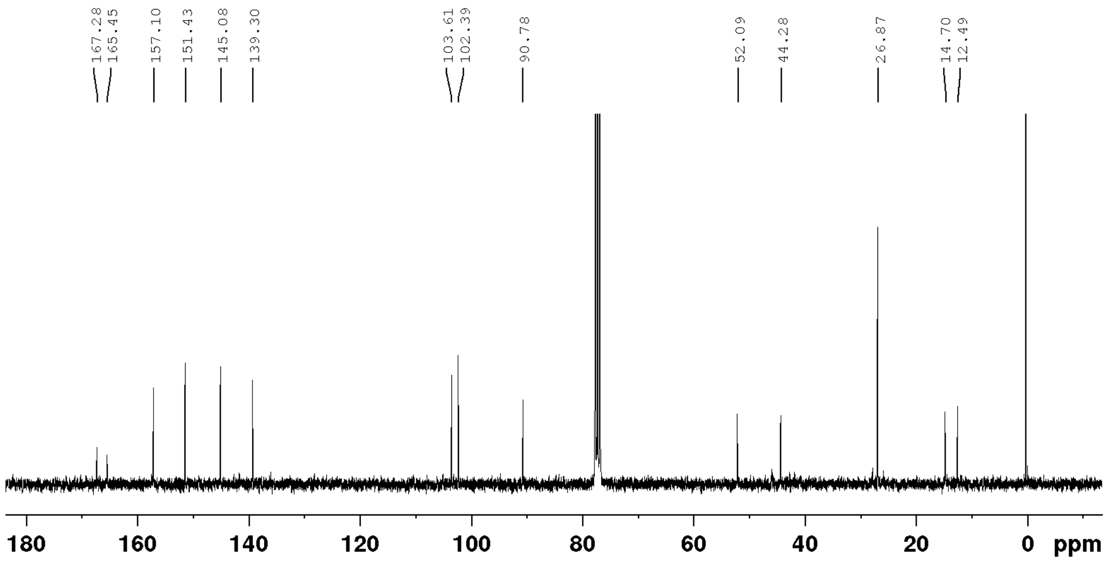

Figure S2. ^{13}C NMR spectrum of DASA-M.

^{1}**H NMR** (300 MHz, $CDCl_3$) δ 11.43 (s, 1H), 7.30-7.25 (d, *J* = 12.3 Hz, 1H), 7.05 (s, 1H), 6.75-6.71 (dd, *J* = 12.43, 1.29, 1H), 6.10-6.02 (t, *J* = 12.35, 1H), 3.53-3.46 (q, *J* = 7.24 Hz, 4H), 1.71 (s, 6H), 1.37-1.22 (m, 6H); ^{13}**C NMR** (75 MHz, $CDCl_3$) δ 167.3, 165.5, 157.1, 151.4, 145.1, 139.3, 103.6, 102.4, 90.8, 52.1, 44.3, 26.87, 14.7, 12.5.

3. Fluorescence Enhancement of DASAs by CDs and CB[7]

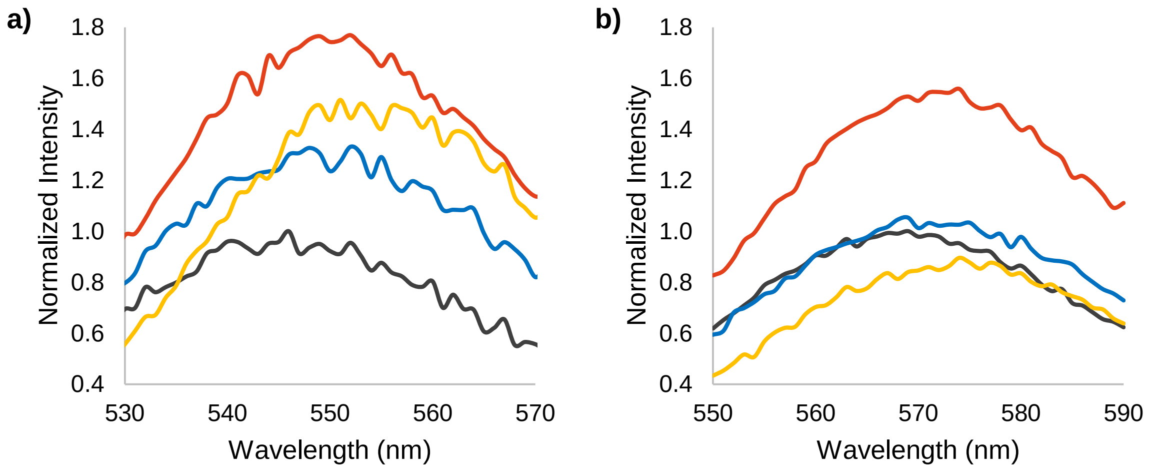

Figure S3. Normalized emission intensity of (**a**,**b**) DASA-M and DASA-B with no host (−), 20 mM HP-α-CD (−), 20 mM HP-β-CD (−), or 20 mM HP-γ-CD (−), showing that encapsulation by HP-γ-CD results in the greatest fluorescence enhancement for DASA-M and DASA-B.

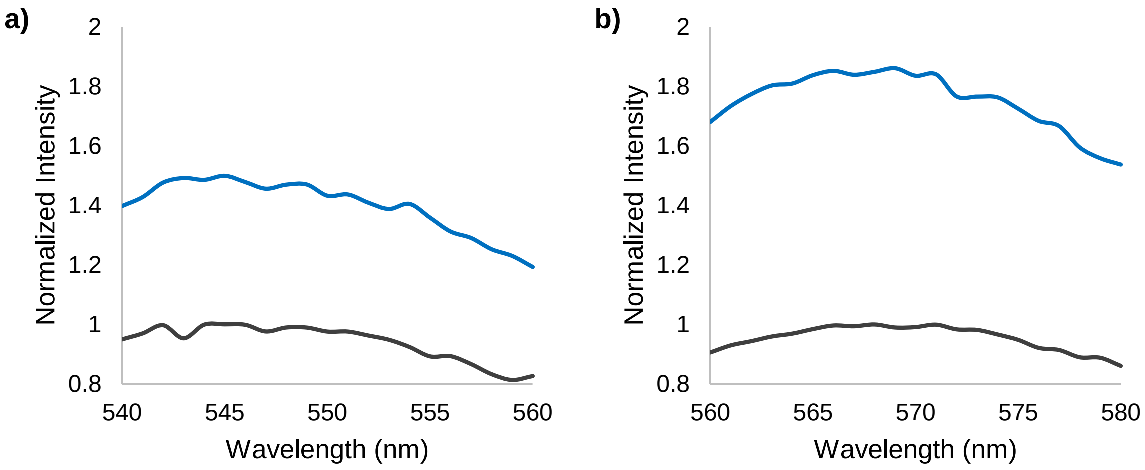

Figure S4. Normalized emission intensity of (**a**,**b**) DASA-M and DASA-B with no host (−) or 20 µM CB[7] (−).

4. Solvent- and Host-Based Negative Solvatochromism

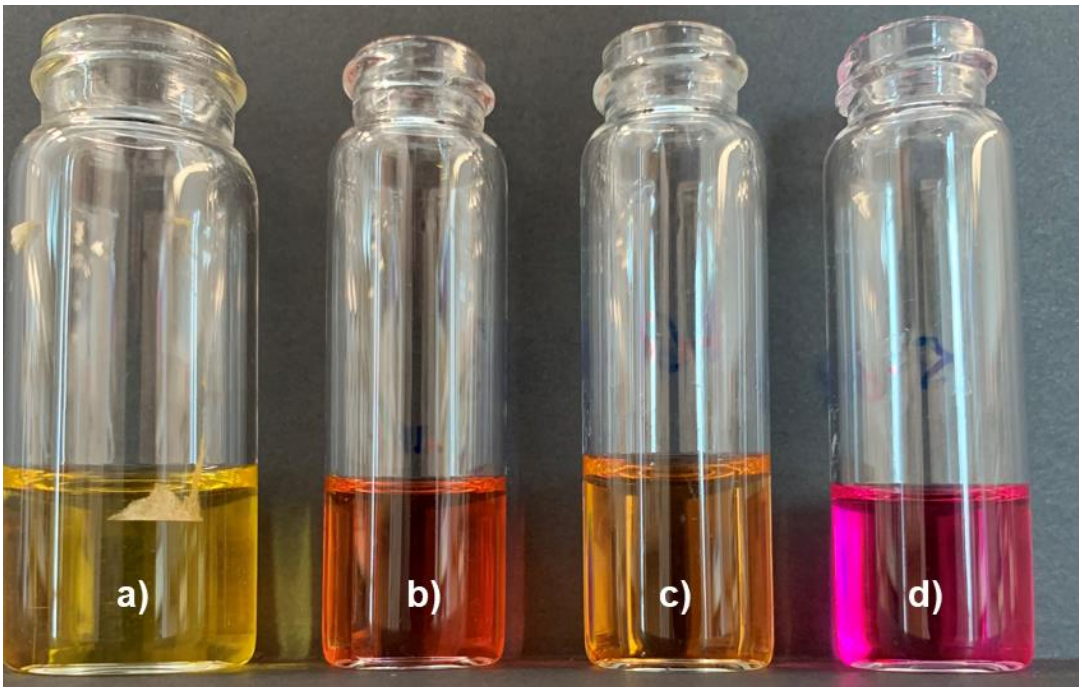

Figure S5. Solvent-based negative solvatochromism: (**a**) DASA-M in H_2O; (**b**) DASA-M in EtOH; (**c**) DASA-B in H_2O; (**d**) DASA-B in EtOH.

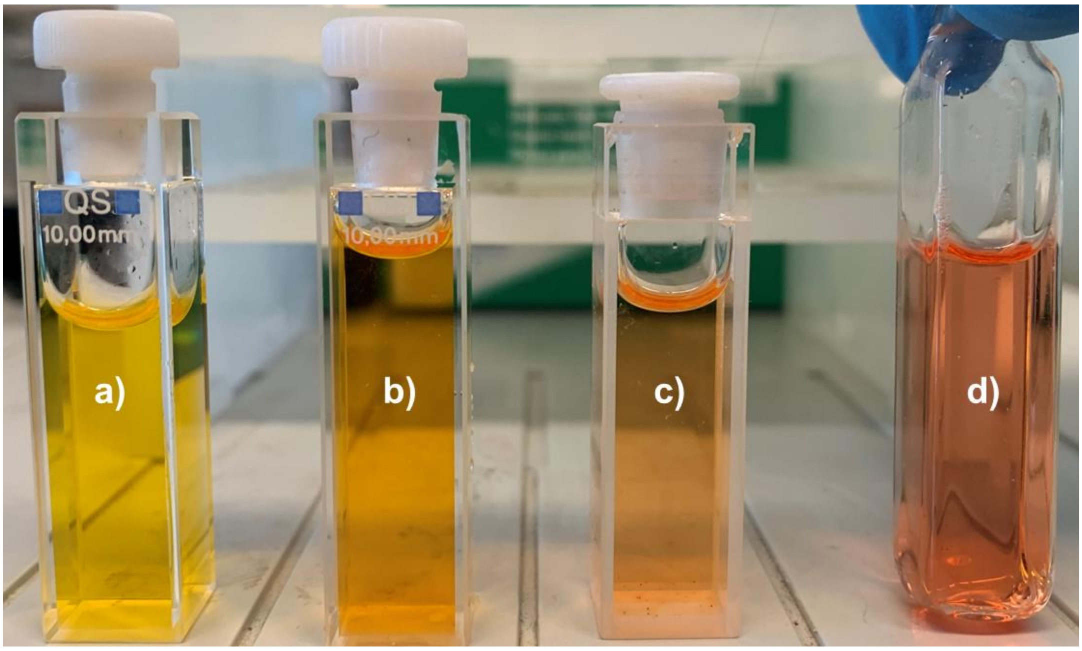

Figure S6. Host-based negative solvatochromism: (**a**) DASA-M in H_2O; (**b**) DASA-M in H_2O with 20 mM HP-γ-CD; (**c**) DASA-B in H_2O; (**d**) DASA-B with 20 mM HP-γ-CD in H_2O.

References

1. Bigi, F.; Carloni, S.; Ferrari, L.; Maggi, R.; Mazzacani, A.; Sartori, G. Clean synthesis in water. Part 2: Uncatalysed condensation reaction of Meldrum's acid and aldehydes. *Tetrahedron Lett.* **2001**, *42*, 5203–5205.
2. Helmy, S.; Leibfarth, F. A.; Oh, S.; Poelma, J. E.; Hawker, C. J.; Read de Alaniz, J. Photoswitching Using Visible Light: A New Class of Organic Photochromic Molecules. *J. Am. Chem. Soc.* **2014**, *136*, 8169–8172.
3. Helmy, S.; Oh, S.; Leibfarth, F. A.; Hawker, C. J.; Read, d. A. Design and Synthesis of Donor-Acceptor Stenhouse Adducts: AVisible Light Photoswitch Derived from Furfural. *J. Org. Chem.* **2014**, *79*, 11316–11329.
4. Lerch, M. M.; Wezenberg, S. J.; Szymanski, W.; Feringa, B. L. Unraveling the Photoswitching Mechanism in Donor-Acceptor Stenhouse Adducts. *J. Am. Chem. Soc.* **2016**, *138*, 6344–6347.
